# Emerging Schemes for Advancing 2D Material Photoconductive-Type Photodetectors

**DOI:** 10.3390/ma16237372

**Published:** 2023-11-27

**Authors:** Huanrong Liang, Yuhang Ma, Huaxin Yi, Jiandong Yao

**Affiliations:** State Key Laboratory of Optoelectronic Materials and Technologies, Nanotechnology Research Center, School of Materials Science & Engineering, Sun Yat-sen University, Guangzhou 510275, China; lianghr27@mail2.sysu.edu.cn (H.L.); mayh9@mail2.sysu.edu.cn (Y.M.); yihx8@mail2.sysu.edu.cn (H.Y.)

**Keywords:** 2D materials, photodetectors, improvement strategies, photodetection

## Abstract

By virtue of the widely tunable band structure, dangling-bond-free surface, gate electrostatic controllability, excellent flexibility, and high light transmittance, 2D layered materials have shown indisputable application prospects in the field of optoelectronic sensing. However, 2D materials commonly suffer from weak light absorption, limited carrier lifetime, and pronounced interfacial effects, which have led to the necessity for further improvement in the performance of 2D material photodetectors to make them fully competent for the numerous requirements of practical applications. In recent years, researchers have explored multifarious improvement methods for 2D material photodetectors from a variety of perspectives. To promote the further development and innovation of 2D material photodetectors, this review epitomizes the latest research progress in improving the performance of 2D material photodetectors, including improvement in crystalline quality, band engineering, interface passivation, light harvesting enhancement, channel depletion, channel shrinkage, and selective carrier trapping, with the focus on their underlying working mechanisms. In the end, the ongoing challenges in this burgeoning field are underscored, and potential strategies addressing them have been proposed. On the whole, this review sheds light on improving the performance of 2D material photodetectors in the upcoming future.

## 1. Introduction

Photoelectric conversion is the pivotal function of various modern civilian and military applications, including sunlight harvesting [1], medical diagnosis [2,3,4,5], optical communications [6], radiation monitoring [7,8], LiDAR [9], Internet of Things [10], e-skin [11], imaging [12,13,14], artificial photonic nociceptors [15], health monitoring [16], target tracking [17], information encryption [18], etc. Photodetectors represent a family of optoelectronic devices that can convert elusory optical signals into easily processed and modulated electrical signals. Traditional photodetectors are usually prepared based on bulk covalent semiconductors, such as silicon [19], indium gallium arsenic [20], group IIIA nitrides [21], etc. However, with the rapid development of intelligence and automation technologies, the technical standards for photodetectors are becoming increasingly stringent, including the demand for superior flexibility, better portability, a higher integration level, and so on. Due to their inherent structural characteristics, the limitations of traditional materials in terms of photodetection have gradually become prominent, and they thus cannot fully meet the growing demand for next-generation optoelectronic applications.

Two-dimensional layered materials (2DLMs) are denoted as a class of ultra-thin phases in which atomic-scale planar structural units are bonded by weak van der Waals force, while the intralayer atoms are commonly conjugated by strong covalent bonds [22,23,24,25,26,27,28]. In recent years, 2DLMs have attracted widespread attention from researchers worldwide due to their excellent and abundant physical and chemical properties, and these materials have been widely applied in various industries, such as fundamental physics [29,30,31,32,33,34], electronics [35,36,37,38,39,40,41,42], photonics [43,44,45,46,47,48], piezo-phototronics [49], catalysis [50,51,52], batteries [53,54,55,56], energy storage [57], thermal management [58], etc. Due to the high in-plane carrier mobility, self-passivated surface, excellent flexibility, wide availability, good compatibility with the modern microfabrication platform, and thickness/strain-dependent energy band structures, 2DLMs have shown indisputable application prospects in the next generation of photodetection applications [59,60,61,62,63,64]. For example, in 2022, Dodda et al. demonstrated a low-energy-consumption active pixel sensor matrix based on a monolayer MoS_2_ phototransistor array [65]. In another study, Xie et al. achieved an exceptional polarization-perceptual neuro-transistor with reconfigurable anisotropic vision by exploiting the low-symmetry ReS_2_ nanosheet as the light-sensing channel [66]. Remarkably, several proof-of-concept polarization-perceptual applications, such as polarized navigation with reconfigurable adaptive learning abilities and 3D visual polarization imaging, have been experimentally realized. Most recently, Gao et al. constructed a transparent hemispherical optoelectronic array for artificial electronic eyes [67]. Proof-of-concept double-sided imaging has been realized based on two kinds of electronic eye prototypes (concave and convex hemispheres) in a single device configuration. Thus far, researchers have developed numerous photodetectors based on various 2DLMs spanning elemental semiconductors/semimetals [68,69,70,71,72], chalcogenides [73,74,75,76,77,78,79,80,81,82,83], nitrides [84], multi-elemental semiconductors [85,86,87,88,89,90,91,92], etc.

Despite the encouraging development, further breakthroughs of 2DLMs in the field of photodetection have still been confronted with some pivotal challenges. On the one hand, due to the substantially small absorption cross-section caused by the atomic-level out-of-plane dimension, the light absorption of 2DLMs is commonly quite low, which hinders the generation of photocarriers. On the other hand, the lifetime of photocarriers in 2D materials is usually limited, predominantly stemming from the pronounced interface effects and strong quantum confinement, which plagues the accumulation of photocarriers. Therefore, there is still much room for improvement in the photosensitivity of 2DLM photodetectors.

Taking these deficiencies into account, researchers have developed a series of optimization strategies for 2D material photodetectors. However, in the current stage, these improvement strategies have been still relatively scattered. To date, although a handful of reviews have summarized the research progress on improving the 2DLM photodetectors, these studies have usually only focused on an individual kind of improvement strategy, such as light manipulation [93,94]. Currently, there is still a lack of a systematic review regarding the performance improvement in 2DLM photodetectors in multiple perspectives. In order to help researchers in this field to have a comprehensive and in-depth understanding, this review provides a systematic overview on the recently harnessed approaches for improving 2DLM photodetectors, by categorizing them as improvement in crystalline quality, band engineering, interface passivation, light harvesting enhancement, channel depletion, channel shrinkage, and selective carrier trapping, with the focus on how to implement these strategies and their underlying physical principles. In the end, we propose the key challenges that stand in the way of further breakthroughs and potential approaches for solving them. The main purpose of this review is to provide a platform for researchers to have a quick and comprehensive understanding of this fascinating field and to point out the research direction for future optimization of 2DLM photodetectors.

## 2. Figures of Merit of Photodetectors

To quantificationally assess the light-sensing capability of photodetectors, several important performance metrics have been defined, which are illustrated in detail as follows.

### 2.1. Responsivity (R)

Responsivity is a fundamental parameter of photodetectors, which can describe the device’s optoelectronic conversion capability to generate output photocurrent under illumination. It can be extracted by using the following equation:

$$R=\frac{I_{\mathrm{Ph}}}{PA}=\frac{I_{\mathrm{light}}-I_{\mathrm{dark}}}{PA},$$

where *I*_Ph_ is the photocurrent, *P* is the power density of incident light, *A* is the effective sensing area of the photodetector, *I*_light_ is the channel current under illumination, and *I*_dark_ is the channel current in darkness.

### 2.2. External Quantum Efficiency

External quantum efficiency (EQE) is an important parameter describing the efficiency of photodetectors to convert incident photons into photogenerated free carriers (electrons and holes) collected by the electrodes. It can be calculated by using the following equation:

$$\mathrm{EQE}=\frac{hcR}{e\lambda },$$

where *h*, *c*, *e*, and *λ* represent the Planck constant, the velocity of light, the electron charge, and the wavelength of light, respectively.

### 2.3. Detectivity (D*)

Detectivity (D*) is an important indicator describing the sensitivity of a photodetector with respect to noise, and it reflects the capability to identify weak light signals. It can be calculated by the following equation:

$$D^{*}=\frac{\sqrt{A\Delta f}}{\mathrm{NEP}},$$

where Δ*f* is the electrical bandwidth of noise measurement, and NEP is the noise-equivalent power. If shot noise is the predominant noise of a photodetector, detectivity can be estimated according to the following formula:

$$D^{*}=\frac{R\sqrt{A}}{\sqrt{2eI_{\mathrm{dark}}}}.$$

### 2.4. Rise/Decay Time

The speed of photodetectors can be assessed by rise time (*τ*_r_) and decay time (*τ*_d_). Rise time refers to the time duration required for the photocurrent to rise from 10% to 90% of its saturated value after light exposure, while decay time refers to the time duration required for the photocurrent to decrease from 90% to 10% of its saturated value after the illumination terminates.

More details about the figures of merit of 2DLM photodetectors can be found in a recent literature focusing on this topic [95].

## 3. Improvement in Crystalline Quality

As for most 2DLMs, a variety of lattice defects will be easily formed during the invasive material preparation process. For example, the high-temperature synthesis process (e.g., chemical vapor deposition, metal-organic chemical vapor deposition, chemical vapor transport) will inevitably lead to the formation of a large number of atomic vacancies, antisites, and severe interfacial distortion/strain in 2DLMs [96,97,98]. These crystal defects are the strong scattering and recombination centers of photogenerated carriers, which thus will seriously deteriorate the performance of the corresponding electronic and optoelectronic devices. In the past decades, annealing treatment has been proven to be an effective method to improve the crystalline quality of numerous materials [99,100,101], as it provides additional energy and sufficient time for the recovery of the non-ideal lattices. In this consideration, it is a potential approach to improve the performance of 2DLM photodetectors via annealing.

**Figure 1:**
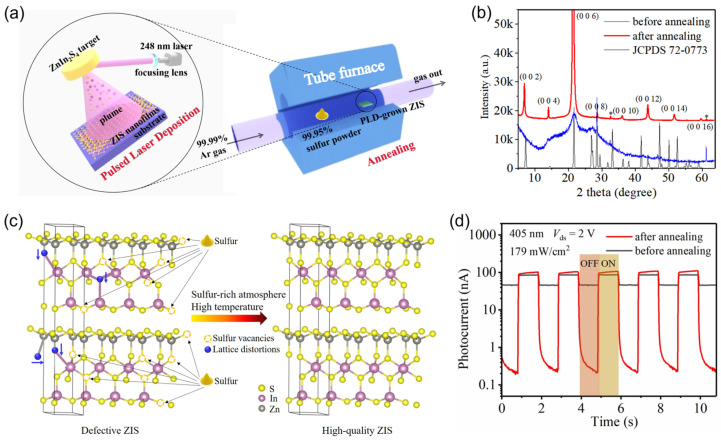
(**a**) Schematic diagram of the two-step fabrication of ZnIn_2_S_4_ nanofilms, including the pulsed-laser deposition and the post-growth high-temperature annealing. (**b**) X-ray diffraction patterns of ZnIn_2_S_4_ nanofilms before annealing (blue line) and after annealing (red line). The star symbols indicate the diffraction signals from the SiO_2_/Si substrate. (**c**) Schematic illustration of the healing of lattice defects of ZnIn_2_S_4_ nanofilm during the post-growth annealing processing. (**d**) Photoswitching curves of the photodetectors based on ZnIn_2_S_4_ nanofilms before annealing (blue line) and after annealing (red line). Reprinted with permission from Ref. [85]. Copyright 2022 Copyright Wiley-VCH (Wehenheim, Germany).

To this end, in 2022, Ye et al. successfully developed the post-deposition annealing technique to improve the performance of the ZnIn_2_S_4_ nanofilm photodetectors prepared by pulsed-laser deposition (PLD, Figure 1a) [85]. In this study, the annealing treatment is conducted at a high substrate temperature of 600 °C, accompanied by the synchronous thermal evaporation of sulfur powder. As shown in Figure 1b, after the high-temperature annealing treatment, the X-ray diffraction peaks of the PLD-derived ZnIn_2_S_4_ nanofilm have been significantly enhanced as compared to the pristine nanofilm, and the full width at half maximum (FWHM) markedly decreases (from 2.08° to 0.3°), indicating a significant improvement in the crystallization quality and a remarkable alleviation of the internal lattice strain. There are two fundamental reasons accounting for the improved crystallinity (Figure 1c): Firstly, the excess sulfur vapor provided by the thermal evaporation of the sulfur powder source can help repair the S vacancies. Secondly, the high-temperature annealing environment can provide sufficient energy to restore the distorted atoms back to the ideal lattice sites. Figure 1d presents the photoswitching curves of the ZnIn_2_S_4_ photodetectors without annealing treatment and with 600 °C annealing treatment under the same working conditions. It is evident that the dark current decreases by ≈2 orders of magnitude after the annealing treatment, which is beneficial for suppressing the standby energy dissipation. This is because the free carriers generated through the thermal excitation of defect states will decrease with the reduction in defect states. Meanwhile, the photocurrent of the annealed device has been increased by several times as compared to that of the original one. This is because both the scattering efficiency and the recombination efficiency of photocarriers will decrease with the reduction in defect states, thereby increasing their transport efficiency and extending their lifetime. As a consequence, the photocurrent (*I*_Ph_) increases according to [102]

*I*_Ph_∝*Nƞμτ* = Δ*nμτ*,

where *N* is the number of absorbed photons, *ƞ* is the conversion efficiency of absorbed photons to photocarriers, *μ* is the carrier mobility, *τ* is the carrier lifetime, and ∆*n* is the carrier concentration of photocarriers. Taking advantage of the improvement in the crystalline quality of photosensitive channels, the optimal responsivity, EQE, and detectivity of the annealed ZnIn_2_S_4_ device have reached 1.4 A/W, 430%, and 9.8 × 10^9^ Jones, respectively.

Since the fundamental principle of improving crystallinity through thermal annealing treatment is largely independent of the specific component/structure of the annealed materials, this strategy exhibits broad universality. In reality, annealing treatment has so far been harnessed to improve the crystalline quality of a series of 2DLMs, such as MoS_2_ [103], SnS_2_ [104], and h-BN [105]. In the future, it can be expected that annealing treatment can be utilized to improve various 2DLM photodetectors. However, it is worth emphasizing that a large number of vacancies will be concurrently generated during the high-temperature treatment [106,107], because atoms readily gain sufficient energy required for escape from the lattice. This phenomenon will be unneglected provided that the annealed material contains elements with high saturated vapor pressure, such as sulfur and selenium. Accordingly, the post-growth annealing treatment usually needs to be performed under a specific gas atmosphere, which can help compensate for the element loss, to ensure the crystallization quality of the 2DLMs.

In addition to thermal annealing, mild plasma treatment has emerged as another strategy to heal the defect states of 2DLMs. For example, in 2022, Li et al. demonstrated a much improved temporal response rate of MoS_2_ photodetectors by exposing them to mild oxygen plasma for an appropriate duration [108]. Impressively, the response/recovery time of the oxygen-plasma-treated MoS_2_ photodetector is approximately three orders of magnitude shorter than that of a pristine MoS_2_ device. Essentially, active oxygen species from the plasma will gradually bond with the vacancies of MoS_2_ in the process of oxygen plasma treatment, which can transform the deep-level defect states into localized defect states. As a result, the shallow-level defect states will dominate the carrier dynamics, thus accelerating the response rate. In a word, this study provides a facile route to improve the performance of 2DLM photodetectors.

## 4. Band Engineering

Due to the strong spacial confinement of free carriers within a 2D plane, as well as the severe interfacial effects, the lifetime of photogenerated carriers in 2DLMs is commonly quite limited. As an example, it is found that the lifetime of photocarriers is dramatically shortened from ≈1000 ps to ≈50 ps as the MoS_2_ nanosheet is thinned down from ten monolayers to one monolayer [109]. On account of the positive dependence of the photoresponse of photoconductive-type photodetectors on the lifetime of non-equilibrium carriers [110], the above peculiarity of 2DLMs is disadvantageous for the implementation of high-performance photodetection.

Band engineering is one of the most effective approaches to regulate the lifetime of photocarriers by modulating the carrier dynamics through band tailoring. For example, time-domain ab initio simulations indicate that tensile strain can extend the lifetime of charge carriers of monolayer WSe_2_ by tailoring the electron-phonon coupling [111]. Therefore, band engineering is one of the potential pathways to improve the performance of 2DLM photodetectors.

**Figure 2:**
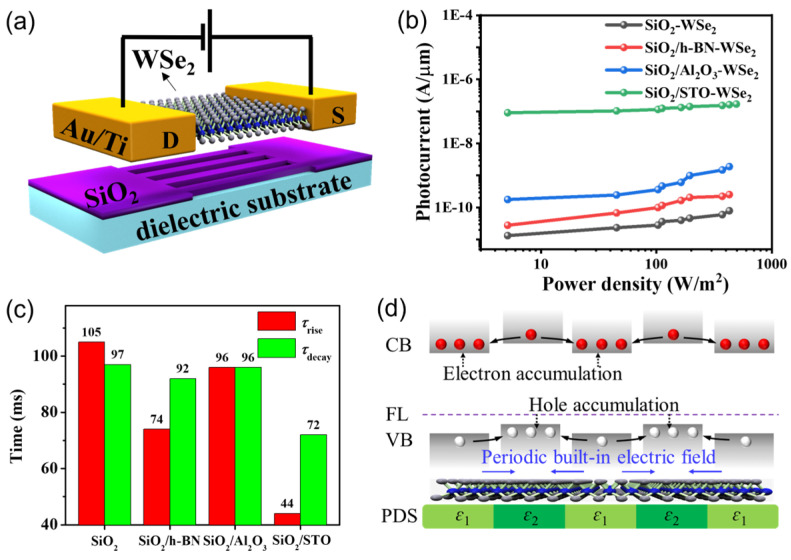
(**a**) Schematic diagram of the PDS-WSe_2_ photodetector. (**b**) Photocurrent of the pristine WSe_2_, SiO_2_/h-BN-WSe_2_, SiO_2_/Al_2_O_3_-WSe_2_, and SiO_2_/STO-WSe_2_ photodetectors as a function of light power density. (**c**) The response time and recovery time of pristine WSe_2_, SiO_2_/h-BN-WSe_2_, SiO_2_/Al_2_O_3_-WSe_2_, and SiO_2_/STO-WSe_2_ photodetectors. (**d**) Energy band diagram and carrier dynamics of PDS-2DLM upon illumination. Reprinted with permission from Ref. [112]. Copyright 2022 Copyright Wiley-VCH (Wehenheim, Germany).

To this end, in 2022, Lu et al. implemented the spacial tailoring of the energy bands of 2DLMs based on a dielectric engineering strategy, which markedly improved the performance of 2DLM photodetectors [112]. In this research work, a series of periodic dielectric structures (PDS), including SiO_2_/h-BN, SiO_2_/Al_2_O_3_, and SiO_2_/SrTiO_3_ (STO), were coupled to the 2D WSe_2_ photosensitive channels (Figure 2a). Remarkably, the photoresponse of the heterostructured devices was significantly improved as compared to the original WSe_2_ photodetector (Figure 2b). In addition, the response/recovery time of the PDS-supported devices also became slightly shorter (Figure 2c). Most notably, it was established that the performance of the PDS-supported device monotonically improves as the periodicity of the PDS structure decreases. Specifically, the optimized SiO_2_/STO-WSe_2_ photodetector achieves a high responsivity, EQE, and detectivity of 89081 A/W, 2.7 × 10^7^%, and 1.8 × 10^13^ Jones, respectively, which enables the reliable identification of weak light as low as 1 pW. In principle, the performance improvement is closely related to the built-in electric fields caused by the PDSs, which can effectively separate the photogenerated electron–hole pairs and significantly extend the lifetime of non-equilibrium photocarriers, thus resulting in a high photogain (Figure 2d). As the periodicity of PDS decreases, the spatial density of the built-in electric fields increases, thus leading to improvement in device performance. More importantly, this improvement strategy was also successfully applied to 2D WS_2_ channels, confirming its broad university. On the whole, this research work achieved the spatial customization of the band structure of 2DLMs through the regulation of the neighboring dielectric environments, opening up a new pathway for the realization of 2DLM homogeneous junctions and the optimization of 2DLM photodetectors.

## 5. Interface Passivation

On account of the substantially high surface-to-volume ratio and the weak electrostatic screening effect of 2DLMs induced by their atomic-level channel thickness, the interface quality plays a crucial role in the performance of 2DLM devices. For example, by conducting a vacuum annealing treatment at 450 °C, Chow et al. determined that the hysteresis in the transfer curve of a PdSe_2_ field-effect transistor nearly disappeared, which was a benefit from the removal of surface adsorbates during the annealing process [113]. In addition to the surface adsorbates, there are many other ingredients on the interface that can degrade the performance of 2DLM devices, including surface fluctuation of substrates, surface defects of substrates, interfacial strain/distortion, interfacial Coulomb impurities, etc. These ingredients are commonly the high-efficiency scattering and recombination centers of photogenerated carriers. In this consideration, optimization of the interface quality can promote carrier migration and reduce Coulomb scattering at the interface to enhance the performance of the 2DLM-based devices.

To address these issues, a variety of investigations have been conducted. For example, in 2021, Zhong et al. constructed a high-performance suspended GaS photodetector by transferring an exfoliated GaS nanosheet onto pre-patterned Au electrodes [114]. Impressively, the device achieves a high responsivity of 1730 A/W, an excellent detectivity of ≈10^12^ Jones, and a short response/recovery time of 3.0/8.3 μs. The competitive photosensitivity is mainly attributed to the circumvention of the negative impacts arising from the interfacial scattering and surface traps from substrates, which is beneficial to fully exert the intrinsic potency of the 2D channel and promote the overall performance. However, the suspended device structure is unreliable, and it is also adverse to large-scale preparation/integration. As a consequence, the long-term stability of the suspended 2DLM photodetectors is a pendent predicament to be addressed. Therefore, alternative strategies are demanded. Passivation of the imperfect substrates is a potential strategy. As an example, Uddin et al. unveiled that graphene on h-BN exhibited a three times higher field-effect mobility as compared to graphene on the SiO_2_ substrate [115], consolidating the tangible potential of substrate passivation.

**Figure 3:**
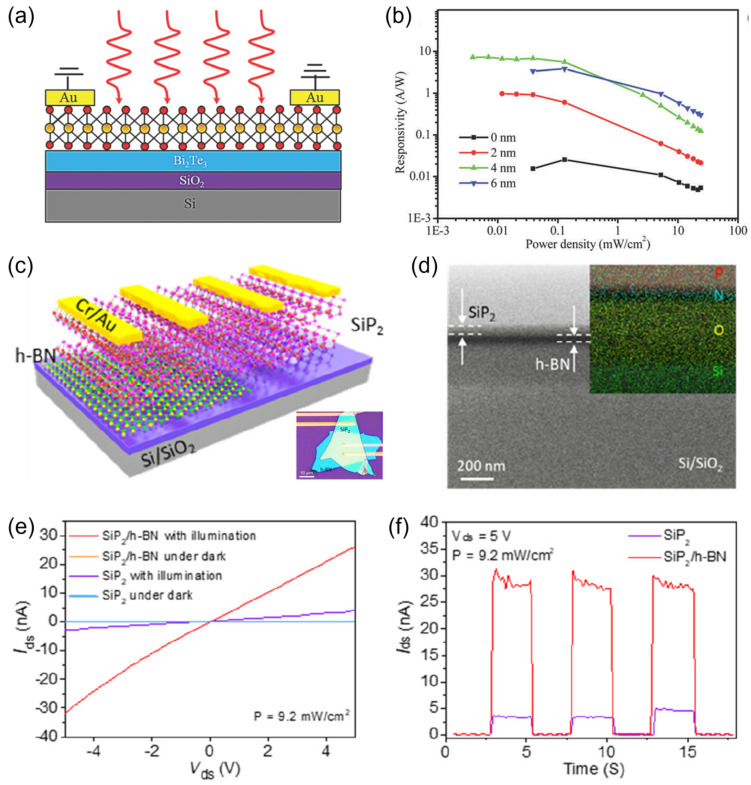
(**a**) Schematic diagram of the WS_2_/Bi_2_Te_3_ photodetector. (**b**) Responsivity as a function of light power density of pristine WS_2_ photodetector and WS_2_/Bi_2_Te_3_ photodetectors with the thickness of the Bi_2_Te_3_ layer to be 2, 4, and 6 nm. Reprinted with permission from Ref. [116]. Copyright 2016, Royal Society of Chemical (London, UK). (**c**) Schematic diagram of the SiP_2_/SiO_2_ and SiP_2_/h-BN devices. The inset shows the corresponding optical microscopic image of the devices. (**d**) Cross-sectional TEM image showing the spacial distribution of the components. The inset shows the element mapping images of P, N, O, and Si. (**e**) *I*_ds_–*V*_ds_ plots of the SiP_2_/SiO_2_ and SiP_2_/h-BN devices in the dark and under illumination. (**f**) *I*_ds_–*t* plots of the devices upon identical periodic illumination. Reprinted with permission from Ref. [117]. Copyright 2023 Copyright American Chemical Society (Washington, DC, USA).

As an example, in 2016, Yao et al. demonstrated an improvement in responsivity by more than two orders of magnitude through passivating the conventional SiO_2_/Si substrate with a Bi_2_Te_3_ layer (Figure 3a,b) [116]. In this study, the Bi_2_Te_3_ passivation layer plays dual roles in terms of improving photosensitivity. On one hand, the *c*-axis-oriented Bi_2_Te_3_ layer can exert an atomically flat and dangling-bond-free surface, which is conducive to reducing the interfacial strain. Accordingly, the WS_2_ layer grown atop exhibits higher crystalline quality as compared to the counterpart WS_2_ layer grown directly on a SiO_2_/Si substrate. On the other hand, Bi_2_Te_3_ has a high dielectric constant, providing efficient electrostatic screening on the Coulomb scattering from charged surface defects/impurities of the SiO_2_/Si substrate. Most recently, Wang et al. harnessed the h-BN nanosheet, an insulating 2DLM, as the dielectric layer for a SiP_2_ nanosheet phototransistor (Figure 3c) [117]. As shown in Figure 3d, the device exhibits a distortion-free interface, suggesting excellent interface quality. As shown in Figure 3e,f, the photocurrent of the SiP_2_/h-BN photodetector is markedly higher than that of the pristine SiP_2_/SiO_2_ counterpart device. By contrast, the dark current values of the two devices are largely comparable. In general, the performance enhancement is attributed to the ideal interface provided by the h-BN layer. On one hand, h-BN is a typical large-bandgap layered vdWM with a self-passivated surface, which can substantially suppress the interfacial strain, as well as the interfacial Coulomb scattering, and thus mitigate the interfacial distortion. On the other hand, the surface of h-BN is atomically flat, which helps to mitigate the scattering effect induced by the substrate’s unevenness. On the whole, this study exemplifies a potential avenue for improving the properties of 2DLM photodetectors without compromising other aspects such as the device reliability, energy dissipation, response rate, signal-to-noise ratio, etc.

It is worth emphasizing that many 2DLM/h-BN heterostructures have been prepared through the traditional transfer methods assisted by polymer stamps [118,119,120], which will inevitably leave residues/contaminations on the surface of the 2D building blocks. For example, the polymethyl methacrylate residue on a transferred MoS_2_ nanosheet can be as high as 35.2% [38]. To address this issue, the in situ construction of 2DLM/h-BN heterostructures is much more favorable. As an example, Fu et al. achieved the direct preparation of MoS_2_/h-BN heterostructures by using a two-step growth method [121]. In brief, few-layer h-BN is initially grown on the Ni–Ga/Mo substrate by chemical vapor deposition using an ammonia borane precursor under atmospheric pressure. Then, H_2_S is introduced into the chemical vapor deposition system to grow MoS_2_ layers on the top of h-BN. Similarly, Chen et al. realized the growth of MoSe_2_/h-BN heterostructures by the initial chemical vapor deposition growth of h-BN and the subsequent molecular beam epitaxy growth of MoSe_2_ [122]. Of note, the interface of the in situ grown heterojunctions is commonly cleaner, sharper, and more intimate as compared to that of the transferred ones, which will thus contribute to further breakthroughs in the performance of 2DLM photodetectors in the future.

Apart from the bottom interface, the top interface of 2DLMs also plays a crucial role in the transport performance of the corresponding electronic devices. As an example, it is revealed that the SnSe field-effect transistor with an h-BN encapsulation layer exhibits substantially reduced hysteresis as compared to a bare SnSe device [123]. In addition, Das et al. demonstrated that the mobility of an Al_2_O_3_-passivated WSe_2_ field-effect transistor is ≈5.5 times that of a bare WSe_2_ device [124].

In 2021, Yang et al. systematically explored the effect of top surface encapsulation on the optoelectronic properties of the MoS_2_ photodetectors [125]. In this study, Al_2_O_3_ was chosen as the encapsulation layer, and it was deposited onto the MoS_2_ channel via atomic layer deposition. Impressively, the responsivity of the Al_2_O_3_-encapsulated MoS_2_ photodetector was ≈2–3 orders of magnitude higher than that of the pristine MoS_2_ device. Moreover, the response rate was also expedited by ≈3 orders of magnitude.

On the whole, surface encapsulation treatment can significantly optimize the top interface of 2DLMs by isolating it from the ambient environment, providing great room for improvement in the performance of 2DLM photodetectors. In the future, new encapsulation strategies from multiple perspectives spanning the encapsulation materials and deposition techniques should be further explored and optimized to provide a new pathway for further improving the performance of 2DLM photodetectors.

Enlightened by the above achievements, it can be reasonably envisioned that excellent device performance can be achieved by simultaneously passivating the top and bottom surfaces of 2DLM photodetectors. As proof, Chen et al. have recently realized an ultrahigh-contrast violet phosphorus (VP) phototransistor by sandwiching the 2D VP channel between dangling-bond-free h-BN layers [72]. Profited from the trap-free heterointerfaces, the VP device exhibited an extremely low dark current of 80 fA and a remarkable on/off ratio of ≈10^5^. Furthermore, a large linear dynamic range of 92.5 dB was realized, and the device remained highly stable over 6000 on/off switching cycles, laying a solid foundation for practical application.

**Figure 4:**
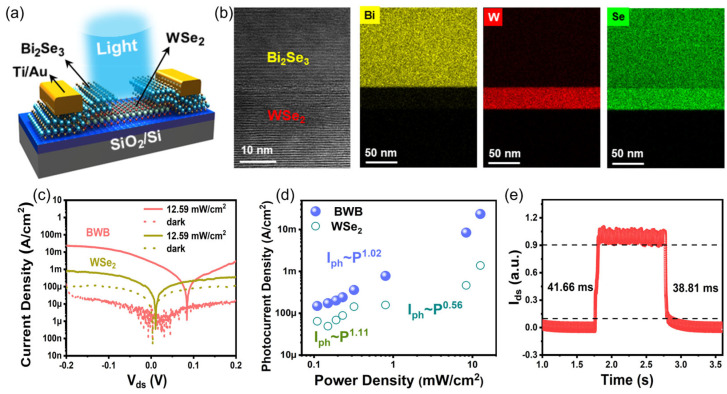
(**a**) Schematic diagram of the Bi_2_Se_3_-WSe_2_-Bi_2_Se_3_ photodetector. (**b**) High-resolution cross-sectional TEM image of the Bi_2_Se_3_/WSe_2_ interface and the corresponding element mapping images of Bi, W, and Se elements. (**c**) *I*-*V* curves of the Bi_2_Se_3_-WSe_2_-Bi_2_Se_3_ and WSe_2_ devices in dark and under illumination with light intensity of 12.59 mW/cm^2^. (**d**) Power-density-dependent photocurrent density of the Bi_2_Se_3_-WSe_2_-Bi_2_Se_3_ and WSe_2_ devices. (**e**) Time-resolved photoresponse of the Bi_2_Se_3_-WSe_2_-Bi_2_Se_3_ device. Reprinted with permission from Ref. [129]. Copyright 2023 Copyright Wiley-VCH (Wehenheim, Germany).

In addition to the channel/air interface and the channel/substrate interface, the electrode/channel interface is also a non-negligible ingredient affecting the device performance. In 2017, for the first time, Yao et al. harnessed topological insulator Bi_2_Te_3_ nanofilms as electrodes for SnSe photodetectors [126]. Impressively, the responsivity and external quantum efficiency of the prepared Bi_2_Te_3_-SnSe-Bi_2_Te_3_ photodetector reach 5.5 A/W and 1833%, respectively, far exceeding those of the previously reported SnSe photodetectors with other electrode materials (such as Ag [127], indium tin oxide [128]) as electrodes. Following this success, in 2023, Huang et al. developed 2D Bi_2_Se_3_ as self-passivated electrodes for the amelioration of 2D WSe_2_ photodetectors (Figure 4a) [129]. In this study, topological insulator Bi_2_Se_3_ and WSe_2_ nanosheets were produced by mechanical exfoliation. The Bi_2_Se_3_ nanosheets were firstly patterned into paired electrodes by a focused ion beam and then dry-transferred onto the WSe_2_ nanosheet. As shown in Figure 4b, the Bi_2_Se_3_/WSe_2_ interface is atomically sharp, exhibiting a high degree of lattice orderliness. This benefited from the van der Waals interactions between the channel and the electrode, which can efficiently prevent the occurrence of physical or chemical interactions at the interface, thus preventing the formation of interfacial defects that curtail the transport property of charge carriers. As shown in Figure 4c,d, the photoresponse of the Bi_2_Se_3_-WSe_2_-Bi_2_Se_3_ (BWB) device is nearly one order of magnitude higher than that of a pristine WSe_2_ device under identical light illumination, while the current density of BWB is lower than the WSe_2_ device in darkness, which is conducive to reducing the standby energy consumption and elevating the signal-to-noise ratio. Moreover, as shown in Figure 4e, the rise time and decay time of the BWB device are 41.66 and 38.81 ms, respectively, which are both shorter than those of the WSe_2_ device (49.56 ms/47.08 ms).

Thus far, in addition to topological insulators, many other metallic/semimetallic 2DLMs, such as graphene [130,131], graphite [132], reduced graphene oxide [133], 1T′-WS_2_ [134], TaSe_2_ [135,136], ZrTe_2_ [137], Sb_2_Te_3_ [138], etc., have been validated as highly competitive self-passivated electrode building blocks. These 2DLMs have different work functions, providing rich choices for device design. In the future, these van der Waals building blocks can also be harnessed to improve the performance of various 2DLM photodetectors.

## 6. Light Harvesting Enhancement

As is well known, the atomic-thin nature also leads to the low absorption of 2DLMs, which significantly hinders their development and application. Localized surface plasmon resonance (LSPR) has been reported to be effective in enhancing the light–matter interactions by coupling and trapping freely propagating plane waves into an adjacent semiconductor. To date, researchers have discovered a series of optical antenna materials with pronounced plasmonic resonance effects, including Au [139], Ag [140], Al [141], Pt [142], Pd [143], etc. Therefore, integrating plasmonic optical antennas with 2DLMs can overcome the notorious disadvantage of insufficient light absorption.

**Figure 5:**
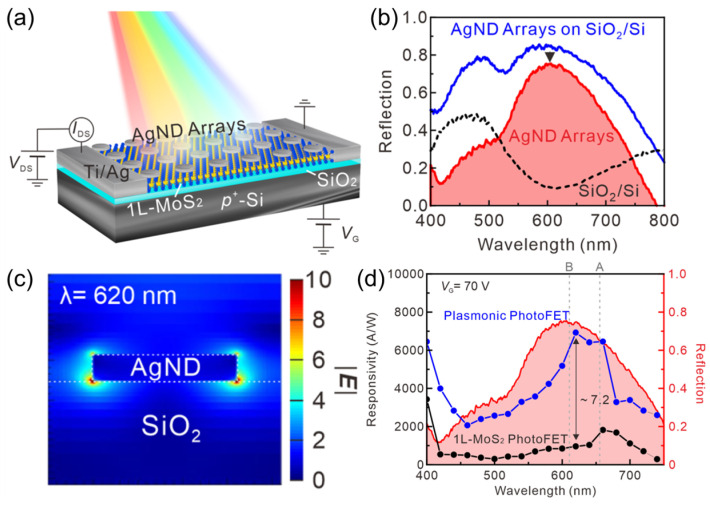
(**a**) Schematic diagram of a monolayer MoS_2_ phototransistor modified with Ag nanodisks (AgNDs). (**b**) Reflection spectra of the bare SiO_2_/Si substrate (black line) and the AgNDs modified SiO_2_/Si substrate (blue line). The red line indicates the effect induced by the localized surface plasmon resonance effect from the AgNDs. (**c**) Spacial distribution of the light field intensity of the cross-section of a AgND on SiO_2_. (**d**) Responsivity as a function of wavelength of pristine MoS_2_ (black dots) and AgNDs/MoS_2_ (blue dots) photodetectors. Reprinted with permission from Ref. [144]. Copyright 2021 Copyright American Chemical Society (Washington, DC, USA).

As proof, in 2021, Lan et al. demonstrated plasmon-enhanced photodetection in a monolayer MoS_2_ phototransistor, which manifested ultrahigh photoresponsivity (Figure 5a) [144]. In this study, silver nanodisk (AgND) arrays were patterned onto the MoS_2_ channel via e-beam lithography and thermal evaporation. As shown in Figure 5b, the plasmonic resonant effect (red curve) contributes a tremendous enhancement of the interactions with light in the range of 500 to 750 nm. Finite difference time domain (FDTD) simulations indicate that this is a benefit from the strong LSPR effect of the AgND antennas, which results in the localized light field intensity (|*E*|) enhancement in the vicinity of the AgNDs (Figure 5c), since the absorbed electromagnetic energy (*P*_abs_) is proportional to the square of the light field intensity according to [145]

Pabs=12ωImεE2,

where *ω* is the frequency of incident light, and Im(*ε*) is the imaginary part of the permittivity. As such, more photocarriers can be excited in the monolayer MoS_2_ channel with the surface modification of the AgND array. Figure 5d presents the spectral photoresponse of the pristine MoS_2_ and AgNDs/MoS_2_ photodetectors. It is clearly determined that the responsivity of the AgNDs/MoS_2_ photodetector is superior to that of the counterpart MoS_2_ device across a broad spectral range from 400 to 750 nm. Of note, a 7.2-fold enhancement is realized upon resonant light excitation (620 nm). Most recently, Wang et al. have improved the photosensitivity of a monolayer MoS_2_ photodetector by modifying a layer of Au nanoparticles [146]. Importantly, upon 532 nm illumination, an 11-fold enhancement in photocurrent was realized. Furthermore, the response rate remained unaffected with the optical antennas. In another study, Li et al. found that double-layered plasmonic optical antennas exhibited a much better enhancement effect for 2DLM photodetectors as compared to the single-side ones [147]. Specifically, the photocurrent of the MoS_2_ photodetector sandwiched between two layers of Au nanoparticles is almost three times that of the MoS_2_ photodetector covered on a layer of Au nanoparticles. This study depicts a straightforward paradigm to fully exert the LSPR effect of precious metal optical antennas to enhance the photoresponse of 2DLM optoelectronic devices.

Since the plasmonic resonance effect is predominantly determined by the material and structure of the optical antennas, and it has little relationship with the adjacent photosensitive channels, this improvement scheme can be conveniently applied to other 2DLMs, provided the working band matches the resonance band of the optical antenna. In this consideration, this improvement strategy exhibits broad universality. Thus far, in addition to MoS_2_, researchers have actually improved a series of 2DLM photodetectors by using plasmonic optical antennas, including SnSe_2_ [148], SnS_2_ [149], ReS_2_ [150], InSe [151], etc. On the whole, the integration of optical antennas with a pronounced LSPR effect provides a universal protocol for improving the 2DLM photodetectors without compromising the response rate.

As mentioned above, the current metallic-nanostructure-based plasmonic optical antennas are cost-inefficient, and their plasmonic resonance spectra are commonly restricted to the range of ultraviolet to visible light, limited by the intrinsic properties of these materials [152,153]. Therefore, there is still an imperious demand to further explore new strategies that can improve the light absorption of 2DLM photodetectors without compromising the preparation cost.

**Figure 6:**
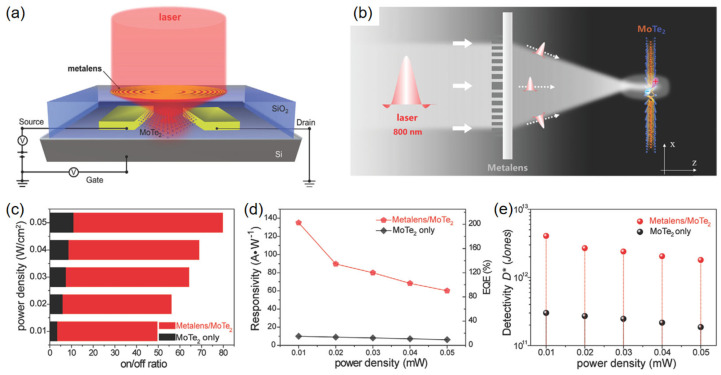
(**a**) Schematic view of a metalens-integrated MoTe_2_ photodetector. (**b**) Schematic illustration of the light focusing by the Fresnel zone plate metalens integrated on the MoTe_2_ channel. Comparison of the (**c**) on/off ratio, (**d**) responsivity, EQE, and (**e**) detectivity between metalens/2H-MoTe_2_ photodetector and intrinsic 2H-MoTe_2_ photodetector under different power densities. Reprinted with permission from Ref. [154]. Copyright 2022 Copyright Wiley-VCH (Wehenheim, Germany).

To overcome this dilemma, in recent years, the integration of micron-lens has been proposed to be a potential method for the performance enhancement of nanomaterial-based optoelectronic devices. As proof, in 2022, Qiao et al. integrated a Fresnel zone plate (FZP) metalens onto 2H-MoTe_2_ photodetectors and successfully improved the optoelectrical properties (Figure 6a) [154]. Essentially, the FZP lens can focus incident light from a relatively large cross-section onto a focal point through a series of concentric circular ring structures, the sizes and positions of which can be precisely designed to achieve customized focal lengths and focusing characteristics. In this study, the TiO_2_ metalens pattern was designed using FDTD simulations and fabricated using standard electron beam lithography and magnetron sputtering techniques. As shown in Figure 6b, the large-area incident light passing through the metalens is fully focused onto the MoTe_2_ channel layer, thus achieving high-efficiency light harvesting. Specifically, the on/off ratio, responsivity, and detectivity of the FZP-integrated MoTe_2_ photodetector are optimized to 49.5, 135 A/W, and 4.05 × 10^12^ Jones, respectively, which are significantly higher than those of the intrinsic MoTe_2_ photodetector (Figure 6c–e). Evidently, the enhanced photosensitive properties are associated with the light focusing enabled by the metalens, which results in high photon utilization efficiency. Because the focusing of light can be flexibly modulated through the regulation of the refractive index/the material and the geometric structure of the focusing lens, this strategy theoretically possesses broad universality, making it well complementary to the conventional plasmonic optical antennas. In the future, this strategy can be expected to be further developed to improve other 2DLM photodetectors operated in the long-wave region, such as graphene [155], Bi_2_Te_3_ [156], black phosphorus [157], PtSe_2_ [158], PdSe_2_ [159,160], PtTe_2_ [161], and so on.

**Figure 7:**
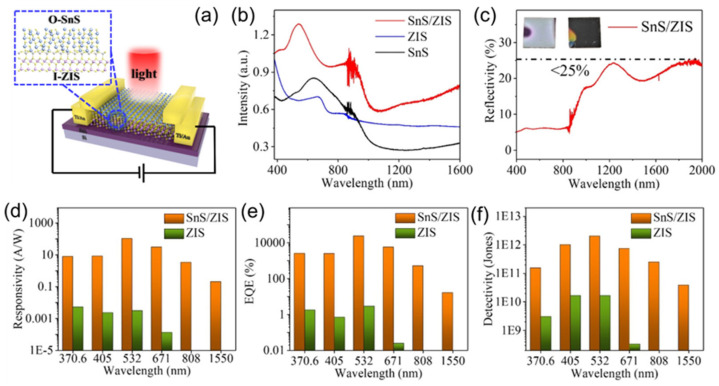
(**a**) Schematic diagram of a V-SnS/I-ZnIn_2_S_4_ photodetector. (**b**) Absorption spectra of the PLD-derived SnS/ZnIn_2_S_4_ (red line), ZnIn_2_S_4_ (blue line), and SnS (black line) nanofilms. (**c**) Reflection spectrum of the PLD-derived SnS/ZnIn_2_S_4_ nanofilm. The digital images of SnS (left) and SnS/ZnIn_2_S_4_ (right) nanofilms are shown in the inset. (**d**–**f**) Summary of responsivity, EQE, and detectivity of the ZnIn_2_S_4_ (green columns) and SnS/ZnIn_2_S_4_ (orange columns) photodetectors upon illumination with various wavelengths. Reprinted with permission from Ref. [162]. Copyright 2022, Royal Society of Chemical (London, UK).

Apart from the plasmonic optical antennas and the light-focusing structures, light capture can also be enhanced through the integration of antireflective structures. To this end, in 2022, Ye et al. successfully improved the photosensitivity of ZnIn_2_S_4_ photodetectors by integrating vertically aligned SnS nanosheets (V-SnS) atop (Figure 7a) [162]. The preparation of V-SnS/ZnIn_2_S_4_ heterostructures is achieved by using a three-step growth method, the PLD growth of ZnIn_2_S_4_, post-deposition annealing, and the PLD growth of V-SnS. Essentially, the rough surface of the annealed ZnIn_2_S_4_ nanofilm induces a higher surface diffusion activation energy of adsorbates than that of the commercial polished SiO_2_/Si substrate. As a consequence, the in-plane migration of the laser-ablated SnS species on the surface of ZnIn_2_S_4_ is markedly restrained, and the out-of-plane oriented V-SnS nanosheets are thus formed. As shown in Figure 7b,c, the 3D-structured SnS nanosheet network on ZnIn_2_S_4_ can induce multiple scattering of incident light, which will reduce the reflectance and significantly enhance the absorbance over the visible to near-infrared range. In addition, SnS and ZnIn_2_S_4_ form a typical type-II staggered band alignment, which benefits the spatial separation of photoexcited electron–hole pairs and markedly extends the lifetime of photocarriers. Benefiting from the synergy of these effects, the responsivity, EQE, and detectivity of the V-SnS/ZnIn_2_S_4_ photodetectors were markedly improved as compared to the pristine ZnIn_2_S_4_ devices (Figure 7d–f).

Up to now, in addition to SnS, a variety of 2DLMs in the vertical forms, including graphene [163], MoS_2_ [164], WS_2_ [165], MoSe_2_ [166], SnS_2_ [167,168], In_2_Se_3_ [169], PtSe_2_ [170], Bi_2_O_2_Se [171], etc., have been experimentally produced. These building blocks have provided a rich palette for the construction of optoelectronic devices, laying a solid foundation for the preparation of high-performance photodetectors used for various wavebands.

Moreover, the enhancement of the light harvesting of 2DLM photodetectors can also be realized by surface modification with suitable photosensitive nanomaterials. For example, in 2022, Peng et al. successfully fabricated high-performance photodetectors by spin-coating PbSe nanocrystals on MoS_2_ nanosheets [172]. On one hand, the PbSe nanocrystals can effectively absorb the incident photons and convert them into photocarriers. On the other hand, the photogenerated electron–hole pairs can be efficiently separated by the built-in electric field at the heterointerface. As a consequence, the responsivity and detectivity of the hybrid PbSe/MoS_2_ device are improved by 512% and 483% as compared to those of a pristine MoS_2_ device, respectively. Most recently, Zhang et al. improved the photoresponse of a 2D SnP_2_S_6_ photodetector by modifying it with a (PEA)_2_PbI_4_ layer through a spin-coating method [173]. The improved photosensitivity is associated with the synergy of the enhanced light absorption by the (PEA)_2_PbI_4_ layer and the promoted electron–hole separation efficiency enabled by the type-II band alignment.

## 7. Channel Depletion

Dark current is one of the most important parameters that characterize the performance of photodetectors, and low dark current is often a significant feature of high-performance photodetectors [174,175,176]. Generally, low dark current can endow the photodetectors with the capability to identify weak optical signals with high accuracy and sensitivity. This is especially important in terms of applications that need to identify faint light or low-intensity signals. In imaging and spectroscopy applications, photodetectors with low dark current can provide high-resolution images or spectra because these devices can distinguish subtle variations in light intensity. In addition, electronic devices with low dark current commonly exhibit low energy consumption, which is of great significance for the implementation of highly integrated electronic chips. However, for practical material preparation, various crystal defects will be inevitably formed in 2DLMs during the synthesis process. For example, the chalcogen elements have relatively high saturated vapor pressure, making it easy for them to escape from the lattice and thus form a large number of chalcogen vacancies during the high-temperature synthesis processes [177,178,179]. These defect states will release a large number of free carriers under thermal excitation, resulting in high dark current. In this consideration, suppressing dark current is an effective means of enhancing the device performance of 2DLM photodetectors.

**Figure 8:**
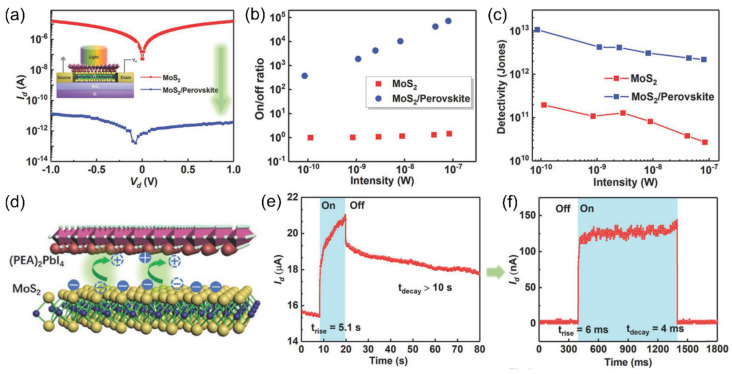
(**a**) *I*-*V* curves of the pristine MoS_2_ device (red line) and the MoS_2_/(PEA)_2_PbI_4_ hybrid PwER device (blue line). The inset shows the schematic diagram of the side view of device structure. (**b**) On/off ratio and (**c**) detectivity as a function of light power of the MoS_2_ device (red dots) and the MoS_2_/(PEA)_2_PbI_4_ hybrid device (blue dots). (**d**) Schematic illustration of the electron transfer process at the MoS_2_/(PEA)_2_PbI_4_ interface. (**e**,**f**) A single photoswithcing curve of the MoS_2_ device and the MoS_2_/(PEA)_2_PbI_4_ hybrid device, respectively. Reprinted with permission from Ref. [180]. Copyright 2020 Copyright Wiley-VCH (Wehenheim, Germany).

To this end, in 2020, Wang et al. reported a MoS_2_ photodetector with suppressed dark current by employing (C_6_H_5_C_2_H_4_NH_3_)_2_PbI_4_ ((PEA)_2_PbI_4_), a 2D halide perovskite, as the electron reservoir [180]. The (PEA)_2_PbI_4_ layer was prepared onto the MoS_2_ nanosheet using a straightforward spin-coating method. As shown in Figure 8a, compared with a pristine MoS_2_ device, the dark current of the hybrid MoS_2_/(PEA)_2_PbI_4_ device was decreased by approximately six orders of magnitude at a bias voltage of 1 V. Benefiting from the suppressed dark current, the on/off ratio and detectivity of the hybrid MoS_2_/(PEA)_2_PbI_4_ photodetector were markedly increased as compared to a pristine MoS_2_ device (Figure 8b,c). Specifically, the on/off ratio was increased from ≈1 to ≈10^5^ upon 80 nW illumination, and the detectivity was significantly increased by about two orders of magnitude from ≈1.96 × 10^11^ to ≈1.06 × 10^13^ Jones under ≈0.1 nW illumination. As shown in Figure 8d, the suppression of dark current is associated with the decrease in the electrical conductivity caused by the migration of electrons from MoS_2_ to (PEA)_2_PbI_4_. In general, MoS_2_ is an n-type semiconductor, while (PEA)_2_PbI_4_ is a p-type semiconductor. A depletion layer will form in the vicinity of the heterointerface provided these two building blocks are in contact, resulting in a decrease in the free carrier concentration. In addition to suppressing dark current, the integration of an electron reservoir can also result in an expedited response rate. As shown in Figure 8e,f, the response/recovery time was significantly shortened from 5.1/>10 s to 6/4 ms after the integration of the (PEA)_2_PbI_4_ layer. This is because the (PEA)_2_PbI_4_ coating layer can passivate the surface of MoS_2_ whilst isolating it from the ambient species (e.g., O_2_, H_2_O).

Overall, this channel depletion scheme enables the reduction in the standby current of metal-semiconductor-metal photodetectors through the neutralization of free carriers by combining materials with the opposite charge polarities, providing a distinctive paradigm for promoting the perception of weak light signals.

## 8. Channel Shrinkage

For the photoconductive-type photodetectors, the channel length plays a dominant role in the time duration for photogenerated carriers to cycle across the channel. In principle, as the channel length increases, the time period required for the photocarriers to traverse will increase. Accordingly, the channel length of 2DLM photodetectors will have an important effect on their photoelectric properties.

**Figure 9:**
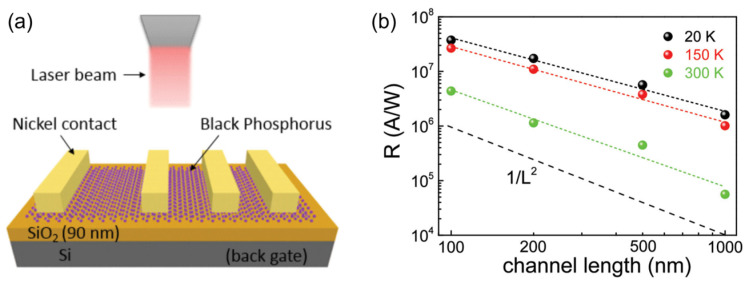
(**a**) Schematic illustration of lateral black phosphorus photodetectors with various channel lengths. (**b**) Responsivity as a function of channel length of black phosphorus photodetectors. Reprinted with permission from Ref. [181]. Copyright 2016 Copyright Wiley-VCH (Wehenheim, Germany).

In 2016, Huang et al. systematically studied the effect of the channel length on the photoelectric characteristics of 2D black phosphorus photodetectors [181]. As shown in Figure 9a, for a convincing comparison, the devices with different channel lengths were constructed on an individual 2D black phosphorus nanosheet. Standard electron beam lithography and electron beam evaporation techniques were exploited to pattern the metal contacts. As shown in Figure 9b, the photoelectric response of the black phosphorus photodetectors is negatively correlated with the channel length. That is, as the channel length decreases, the responsivity of the black phosphorus device monotonically increases. In addition, the responsivity exhibits a distinct inversely proportional dependence on the squared channel length. Specifically, the responsivity of the device with a 100 nm channel reaches ≈4.3 × 10^6^ A/W, which is ≈77 times that of a device with a channel length of 1000 nm. Essentially, the improved photoresponse with decreasing channel length is largely associated with the amplified transverse electric field, which results in shortened transit time (*τ*_transit_) according to

$$\tau_{\mathrm{transit}}=\frac{L^{2}}{\mu V_{\mathrm{ds}}},$$

where *L* is the channel length, *μ* is the carrier mobility, and *V*_ds_ is the source-drain voltage. The photoconductive gain (*G*) is evaluated by

$$G=\frac{\tau }{\tau_{\mathrm{transit}}}=\frac{\mu V_{\mathrm{ds}}\tau }{L^{2}},\;$$

where *τ* is the lifetime of photocarriers. Therefore, the channel shrinkage is conducive to improving the photogain. On the whole, this study highlights that further performance enhancement can be readily achieved by continuously downscaling the channel lengths of 2DLM photodetectors.

Another convenient strategy to shorten the electrode spacing is to construct metal-semiconductor-metal-type photodetectors with vertical device structures, which is enabled by the dangling-bond-free surface of 2DLMs. As proof of the potency of this scheme, in 2016, Massicotte et al. demonstrated ultrafast photodetectors based on the graphene/WSe_2_/graphene heterostructures [182]. In this research work, the vertical van der Waals structures were prepared through a typical dry transfer technique. Graphene was used as electrodes on account of the merits including excellent electron transport capability and high light transmittance. It was determined that the response time of the devices is positively correlated with the channel length (i.e., the thickness of the WSe_2_ nanosheet). Specifically, an ultrashort response time down to 5.5 ps is realized at a channel length of 2.2 nm. Most recently, He et al. prepared a sandwiched graphene/CuBiP_2_Se_6_/graphene photodetector and demonstrated outstanding photosensitivity [88]. Impressively, the responsivity of the vertically structured CuBiP_2_Se_6_ device was several orders of magnitude higher than that of the parallelly structured one. Specifically, upon 405 nm illumination, a high responsivity of 4.9 × 10^4^ A/W and an excellent detectivity of 1.14 × 10^13^ Jones were achieved. Of note, the vertically structured 2DLM devices enabled the implementation of atomic-scale channel lengths without the demand for cutting-edge microfabrication techniques, providing a promising and cost-efficient paradigm for further breakthroughs in optoelectronic performance.

On the whole, since the channel shrinkage strategy relies only on the geometric scale of the devices, this protocol theoretically exhibits broad universality. Thus far, through channel shrinkage, researchers have improved a series of 2DLM photodetectors, including WS_2_ [183], GaSe [184], etc.

## 9. Selective Carrier Trapping

Selective carrier trapping refers to the spacial localization of a single type of photogenerated carriers (electrons or holes) through unique material/device designs, whilst leaving the other type of photocarriers freely migrating across the photosensitive channel [185]. Since the photoexcited electrons and holes are spacially separated, the lifetime of photocarriers can be markedly extended. As a consequence, selective carrier trapping can theoretically be capitalized to improve the photosensitivity of 2DLM photodetectors.

**Figure 10:**
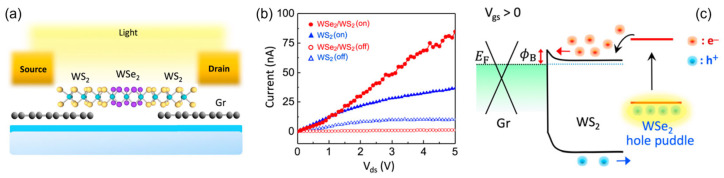
(**a**) Schematic diagram of a WS_2_/WSe_2_ photodetector. (**b**) I–V curves of the WS_2_ photodetector (triangles) and WS_2_/WSe_2_ photodetector (circles) in dark and under illumination. (**c**) Energy band diagram illustrating the carrier dynamics of the WS_2_/WSe_2_ photodetector. Reprinted with permission from Ref. [186]. Copyright 2022 Copyright American Chemical Society (Washington, DC, USA).

As an example, In 2020, Tsai et al. demonstrated the improvement in the photosensitivity of the WS_2_-based photodetectors by embedding dispersive WSe_2_ charge puddles into the WS_2_ channel (Figure 10a) [186]. The implantation of WSe_2_ charge puddles is realized by a two-step process, the initial O_2_ plasma treatment followed by a chemical vapor deposition process which can selectively transform WO*_x_* into WSe_2_. As shown in Figure 10b, in the dark condition, the WSe_2_/WS_2_ photodetector exhibits much lower drain current as compared to a pristine WS_2_ device. This is because the dispersive WSe_2_ puddles serve as the scattering centers, thereby lowering the electron mobility and thus suppressing the channel current. By contrast, a profound enhancement in photocurrent by a factor of ≈3 upon 532 nm illumination is revealed in the WSe_2_/WS_2_ photodetector. To have an in-depth insight, Figure 10c illustrates the carrier dynamic process of the WSe_2_/WS_2_ heterostructure upon light illumination. After light excitation, the photogenerated electron-hole pairs in both WS_2_ and WSe_2_ are efficiently separated by the interfacial built-in electric field at the WSe_2_/WS_2_ p-n junctions as well as the type-II band alignment, where electrons and holes are driven to WS_2_ and WSe_2_, respectively. With the advantage of the spacial separation of the photogenerated electrons and holes, the photoexcited electrons can recirculate across the WS_2_ channel for multiple cycles prior to recombination with the holes trapped in the WSe_2_ puddles, thereby resulting in a large photogain. Furthermore, the WSe_2_ puddles with trapped holes serve as the local gate to the WS_2_ channel, thus further improving the electron concentration, which is well known as the photogating effect. Specifically, upon illumination with a light intensity of 0.04 mW/cm^2^, a high responsivity of 1.7 A/W is achieved, which is nearly an order of magnitude higher than that of a pristine WS_2_ photodetector under the same biasing condition.

On the whole, by selectively trapping a single type of photocarriers to achieve spatial dissociation of photogenerated electrons and holes, the encounter probability of photoexcited electrons and holes can be markedly reduced, thereby significantly extending the lifetime of the non-equilibrium carriers and providing new ideas for improving the performance of 2DLM photodetectors. Thus far, in addition to forming heterogeneous puddles, various strategies have been harnessed to induce selective carrier trapping, including integration of floating channels [187,188,189,190,191], introduction of lattice defects [192], surface modification of quantum dots/metal nanoparticles [75,193,194], surface oxidation [195], etc. In the future, these techniques can be developed for various 2DLM photodetectors to achieve further performance breakthroughs.

## 10. Conclusions

By virtue of the widely tunable band structure, self-passivated surface, pronounced electrostatic tunability, excellent flexibility, and high light transmittance, 2DLMs have proven as potential transformative building blocks toward developing the next-generation photodetectors. Nevertheless, the weak light absorption, limited carrier lifetime, and severe interfacial effects due to the atomically thin nature of 2DLMs have severely plagued the further breakthrough of the photosensitivity of the corresponding 2DLM photodetectors. In the past decade, a host of improvement methods for 2D material photodetectors have been explored. To explicitly sort out the extensive subjects in this burgeoning research field, this review provided a systematical summarization regarding the latest research progress in improving the performance of 2DLM photodetectors by categorizing them as improvement in crystalline quality, band engineering, interface passivation, light harvesting enhancement, channel depletion, channel shrinkage, and selective carrier trapping, with the elaboration on how to implement these schemes and their underlying physical mechanisms. On the whole, this review can serve as a guidepost for the researchers working on improving the performance of 2DLM photodetectors in the near future.

## 11. Outlook

### 11.1. Developing Novel Optimization Strategies

Currently, although the effectiveness of the previously reported optimization strategies has been preliminarily validated, their practical application has still suffered from certain limitations. For example, the integration of plasmonic optical antennas can significantly enhance the light absorption of adjacent 2DLMs in the resonant spectral range. However, generally, traditional metallic nanostructure plasmonic optical antennas can only generate plasmonic resonance in the ultraviolet to visible light range. In this consideration, these materials are, as a rule, inapplicable for long-wave infrared photodetectors. In this consideration, there is an urgent need to develop novel optical antennas for the infrared bands to improve the performance of narrow-bandgap 2DLM photodetectors, in order to satisfy the numerous demands of different applications. Recently, a plethora of non-noble materials, such as Sn: In_2_O_3_ [196], Cu/In: CdO [197], Te-doped Si [198], etc., have demonstrated pronounced plasmonic resonance characteristics in the long-wave infrared spectral range, providing new opportunities for improving the performance of 2DLM infrared photodetectors without compromising the production cost.

### 11.2. Integration and Synergy of Multiple Improvement Strategies

In the preceding endeavors, the vast majority of improvement studies only utilize an individual optimization strategy, which can usually only address a portion of the shortcomings of 2DLMs. For example, the integration of optical antennas is mainly aimed at overcoming the predicament of weak light absorption of 2DLMs. Interfacial passivation engineering is usually aimed at overcoming the serious challenges of carrier scattering or recombination induced by interfacial imperfections. Currently, there is still a lack of device optimization strategies that can simultaneously overcome the multiple deficiencies of 2DLMs. To overcome this dilemma, a potential strategy is to integrate multiple optimization solutions simultaneously in a single device. For example, localized light field enhancement is usually achieved by integrating optical antennas at the top of a 2DLM photosensitive channel [144], while substrate surface passivation is carried out at the bottom of the photosensitive channel [116]. Therefore, these two optimization strategies can theoretically be adapted simultaneously to one device. The integration of multiple optimization strategies provides a new pathway for further breakthroughs in the performance of 2DLM photodetectors.

### 11.3. Promoting Future Applications of 2DLM Photodetectors

Even though the performance of well-designed 2DLM photodetectors meets the standards of practical applications, their commercialization is still confronted with some potential challenges. On one hand, most of the previous studies have focused on constructing a few prototype devices. Nevertheless, the on-chip integration of device arrays is indispensable for achieving powerful functionality [199,200,201], which is relatively less explored. Importantly, a variety of techniques, including molecular beam epitaxy [202,203], chemical vapor deposition [204,205], pulsed-laser deposition [206], atomic layer deposition [207], etc., have by far proven as reliable approaches for preparing large-area 2DLMs. Therefore, in the future, more research enthusiasm should be devoted to developing these techniques for preparing various high-performance 2DLM optoelectronic chips. Beyond this, customizing the thickness of 2DLMs is also extremely challenging, especially in terms of the synthesis of monolayer 2DLMs, since the lateral growth and vertical growth of materials will usually proceed simultaneously. Fortunately, this challenge can be potentially overcome through a two-step growth method separating the nucleation and lateral growth processes [208]. In principle, nucleation is firstly carried out at a relatively low substrate temperature, followed by increasing the substrate temperature above the critical available temperature for nucleation to suppress the vertical growth in the following process. Due to that the edges of the nucleus can still capture precursor species through strong covalent bonds, the lateral growth can still proceed under relatively high temperature. As such, the deposition of monolayer 2DLMs can be achieved. On the other hand, it is important to develop novel optoelectronic applications based on the unique characteristics of 2DLM devices to further expand their application scope. For example, 2DLMs possess excellent flexibility, making them suitable for developing wearable optoelectronic devices for a wealth of functionalities (e.g., health monitoring [209], e-eye [210], ultraviolet radiation warning [7,211]). In addition, many 2DLMs exhibit pronounced in-plane anisotropy, and they can thus be applied to polarized photodetectors, enabling emerging optoelectronic applications such as multiplexing optical communications [212], enhanced contrast imaging [213], etc.

## Data Availability

Not applicable.
